# Use of Suctioning during Newborn Resuscitation and Its Effects on Heart Rate in a Low-Resource Setting, Tanzania

**DOI:** 10.3390/children10091540

**Published:** 2023-09-12

**Authors:** Carolyn Purington, Joar Eilevstjønn, Ingvild Dalen, Anita Yeconia, Ladislaus Blacy, Estomih Mduma, Ingunn Haug, Kari Holte, Catherine Chang, Jeffrey Perlman, Hege Ersdal

**Affiliations:** 1 Department of Population Medicine, Harvard Pilgrim Health Care Institute, Boston, MA 02215, USA; carolyn.purington@gmail.com; 2 Strategic Research, Laerdal Medical, 4007 Stavanger, Norway; joar.eilevstjonn@laerdal.com (J.E.); ingunn.haug@laerdal.com (I.H.); 3 Department of Research, Stavanger University Hospital, 4019 Stavanger, Norway; ingvild.dalen@sus.no; 4 Haydom Lutheran Hospital, Haydom 9000, Tanzania; yeconia@gmail.com (A.Y.); ladislaus.blacy@haydom.co.tz (L.B.); estomduma@gmail.com (E.M.); 5 Department of Paediatrics and Adolescence Medicine, Østfold Hospital Trust, 1714 Grålum, Norway; kari.holte@so-hf.no; 6 Weill Cornell Medicine, Department of Pediatrics, Division of Neonatology, New York, NY 10065, USA; catlchang@gmail.com (C.C.); jmp2007@med.cornell.edu (J.P.); 7 Faculty of Health Sciences, University of Stavanger, 4021 Stavanger, Norway; 8 Department of Anesthesia, Stavanger University Hospital, 4019 Stavanger, Norway

**Keywords:** neonatal resuscitation, oro/nasopharyngeal suction, birth asphyxia, newborn mortality

## Abstract

Suctioning of newborns immediately after birth, as part of delivery room resuscitation, is only recommended if the airway is obstructed. The aim of this study was to describe the use of suctioning during newborn resuscitation among survivors versus those who died within 3 days and potential suction-related heart rate responses and associations to newborn characteristics. This was a retrospective observational study from July 2013 to July 2016 in a referral hospital in rural Tanzania. Research assistants observed and documented all deliveries, newborn resuscitations were video-recorded, and newborn heart rates were captured with a dry-electrode electrocardiogram. Liveborn infants ≥34 weeks gestation who received ventilation and with complete datasets were eligible. All 30 newborns who died were included, and a total of 46 survivors were selected as controls. Videos were annotated and heart rate patterns were observed before and after the suction events. Suctioning was performed more frequently than recommended. No differences were found in suctioning characteristics between newborns who died versus those who survived. In 13% of suction events, a significant heart rate change (i.e., arrhythmia or brief/sustained >15% fall in heart rate) was observed in relation to suctioning. This represents a potential additional harm to already depressed newborns undergoing resuscitation.

## 1. Introduction

Recent studies suggest that the incidence of suctioning and/or bag-mask ventilation (BMV) at birth is considerable in both high- and low-resource settings. In an urban hospital in Norway, 18% of newborns were brought to the resuscitation table, 5% were suctioned, and 4% received BMV [1]. In rural Tanzania, 16% of all newborns received basic stabilization and/or resuscitation, 12% were suctioned, and 8% received BMV [2].

Various risks associated with suctioning have been reported. Suctioning may induce bradycardia [3,4,5] and delay rises in SpO2 levels [6,7]. Furthermore, prolonged and/or repeated suctioning may delay the initiation of ventilation and interrupt effective ventilation, leading to increased risk of adverse outcomes, including death or prolonged admission [2,8,9,10].

In 2006, the Neonatal Resuscitation Program (NRP) emphasized that suctioning was no longer recommended in all newborns immediately after birth [11]. The World Health Organization’s 2012 Guidelines on Basic Resuscitation [12] also recommended that in the presence of clear amniotic fluid, suctioning should only be performed if the mouth or nose are obstructed and should not be done routinely before positive pressure ventilation. International Liaison Committee on Resuscitation (ILCOR) guidelines from 2020 [13] recommended avoiding routine suctioning, even in the presence of meconium-stained amniotic fluid. This recommendation was supported by a 2022 systematic review that found no benefits of suctioning [14].

Helping Babies Breathe (HBB) is a training program in newborn stabilization and resuscitation for low-resource settings [15]. Similar to other international guidelines, the HBB curriculum recommends suctioning only if indicated, e.g., to clear the airways due to obstruction by meconium or mucus [16]. However, even after HBB training, suctioning is documented to be vastly overused [3,5,9,17,18], likely reflecting a widespread practice [19].

The objectives of this study were to describe the incidence and use of suctioning in a low-resource setting and characterize potential suction-related heart rate (HR) responses during newborn resuscitation among survivors compared to newborns who died within 3 days of birth.

## 2. Materials and Methods

This was a retrospective observational study conducted from July 2013 to July 2016 at Haydom Lutheran Hospital (HLH), a referral hospital in rural Northern Tanzania. HLH is a 400-bed hospital providing comprehensive emergency obstetric care and basic emergency newborn care to a population of approximately 500,000 people, with a referral area of approximately 2 million people. HLH contains six labor rooms and one operating theater for cesarean sections, performing approximately 3500–5000 deliveries per year.

Midwives attended routine vaginal deliveries and were the primary providers of neonatal resuscitation, with doctors on call 24 h. All midwives participated in individual skills training through the HBB training program with varying frequency. This study was part of the Safer Births [20] project on the stabilization and resuscitation of newborns in a low-resource setting.

### 2.1. Data Collection

Non-medical research assistants (*n* = 16) were trained to observe and document deliveries and newborn management data (obstetric history, perinatal course and outcomes). Research assistants worked in three shifts to observe every delivery. Newborn resuscitations were video-recorded, and Laerdal Newborn Resuscitation Monitors (LNRMs, Laerdal Global Health, Stavanger, Norway) were used to capture and store HR and ventilation data. LNRMs were installed above each resuscitation table and consisted of a dry-electrode electrocardiogram (ECG) sensor for HR detection, a sensor between the mask and bag capturing flow, pressure, and expired carbon dioxide (CO_2_), and a visual display showing newborn HR. Video cameras were mounted above each resuscitation area to record the newborns and the hands of the provider(s).

LNRM data and videos were downloaded from each delivery room on a weekly basis. Videos and LNRM data were checked by two quality controllers to ensure that all data matched the observational data for each patient, prior to being uploaded to the server. Once files were received on the server, co-author J.E. reviewed each video and LNRM data for accuracy and quality.

### 2.2. Subjects

All live newborns at ≥34 weeks gestational age (GA) who were not crying and received basic resuscitation (defined as additional stimulation and/or suctioning) and/or ventilation, and with complete datasets during the study period, were eligible for enrollment into this study. Stillbirths were excluded. GA was based on self-reports of the last menstrual period and distance from symphysis pubis to fundus on admission. Term GA at HLH was defined as ≥36 weeks. Normal-outcome newborns were defined as surviving >24 h without detected difficulties and were allowed to leave the hospital at 24 h. Admission criteria to the neonatal ward included five-minute Apgar score <7, fever (>38 °C), and signs of respiratory compromise. Available NICU care included antibiotics, supplemental oxygen via nasal cannula, phototherapy, and intravenous fluids. No mechanical ventilation or continuous positive airway pressure were available.

All newborns ≥34 weeks GA who died within 3 days of birth with complete datasets were included (*n* = 30). A group of 30 survivors was randomly selected. As the survivors’ initial HR was higher than the initial HR of those who died, 16 more survivors with low initial HR were randomly selected and added (via random permutation of indices of all applicable cases). Thus, a total of 46 survivors were selected as controls.

### 2.3. Development of the Annotation Protocol

Four independent annotators (C.P., I.H., C.C., K.H.) developed the annotation protocol. The annotators included two physicians (C.C., K.H.), a product development engineer (C.P.), and a human factors engineer (I.H.).

The ELAN program (https://tla.mpi.nl/contact/ (accessed on 1 May 2023), Nijmegen, The Netherlands) was used to describe the following variables: stimulation, suctioning, covering of newborn (to provide warmth), and description of other resuscitation measures (e.g., chest compressions, use of medications, etc.). Interventions were recorded as one continuous intervention as long as pauses were less than 5 s. Specifically, one suction event (SE) was defined as a period of repetitive suctioning with no pause greater than 5 s. Periods of repetitive suctioning with a pause >5 s would be considered 2 SEs. Ten test videos were independently annotated by three annotators in separate ELAN files. Annotations were compared and iterative refinements to the annotation protocol were made until an agreement was reached.

### 2.4. Video Annotations

Video recordings of 30 newborns that died within 3 days and 46 matched survivors were reviewed. Each study video was randomly assigned to two independent annotators for review (one physician and one engineer). Reviewers were blinded to HR, newborn outcome, and observational data at the time of annotations. Following the annotation of all 76 videos, annotations were processed for agreement using Matlab (The Mathworks Inc., Natick, MA, USA). Agreement was considered sufficient when annotations matched ≥80% for the entire resuscitation. Videos with <80% of annotations in agreement were reviewed by the whole group. Each episode of “disagreement” was discussed, and consensus established. Each intervention with obscured view was also reviewed as a group. If interventions were not clearly seen, this was noted.

### 2.5. Suction Annotations

For this substudy, C.P. performed additional detailed annotations of the observed SEs within a limited timeframe (420 s). The videos were annotated for type of suction (Laerdal Penguin reusable bulb suction device or suction catheter) and number of suction insertions in mouth or nose.

### 2.6. Heart Rate and Ventilation Extraction

Ventilation and HR data in the time period around a SE were extracted from the LNRMs. All cases with complete LNRM recordings of HR, ECG, ventilation parameters and stimulation up to 20 s before and after each SE were reviewed manually by J.E. and H.L.E. to assess whether there was a suction-related HR change or not. The HR pre and post each SE were defined as the median of five heart rates preceding the start of and following the cessation of each SE (up to 20 s). A suction-related HR change was coded as an arrhythmia or brief/sustained fall in HR > 15% of starting HR prior to SE, which occurred during and/or within 20 s after the SE, in the absence of concurrent ventilation.

### 2.7. Statistical Analyses

Analyses were performed using SPSS 22, PSPPIRE 1.4.1, and Stata 17.0. Descriptive statistics are presented as counts with percentages, means ± standard deviation (SD) or medians (quartiles) according to distribution. For parameters with repeated observations per newborn, means or medians of individual means over repetitions are presented. Likelihood ratio test *p*-values from regression analyses (linear regression for continuous and logistic regression for categorical dependent variables) without and with adjustment for first HR were used to compare subpopulations unless otherwise stated. In case of severely skewed residuals from linear regression, we used quantile (median) regression; and finally, due to convergence issues with quantile regression, left-skewed variables were reversed and analyzed in log linear models with robust standard errors. Changes in HR were tested for statistical significance using a one-sample Wilcoxon signed-rank test. Flexible modeling using piecewise polynomial functions (splines) was utilized to illustrate nonlinear probability curves for changes in HR in relation to different predictor variables. Specifically, restricted cubic spline (with three knots at default locations) logistic regression fitted with standard errors allowing for clustering was used. The linearity of the effect on log odds was tested using a Wald test, and assumed if *p* > 0.05, in which case odds ratios (OR) with 95% confidence intervals (CI) were presented. For models with statistically significant nonlinearity, we reported chi-square *p*-values for overall fit of model and presented plots of predictions with 95% confidence bands.

## 3. Results

**Figure 1:**
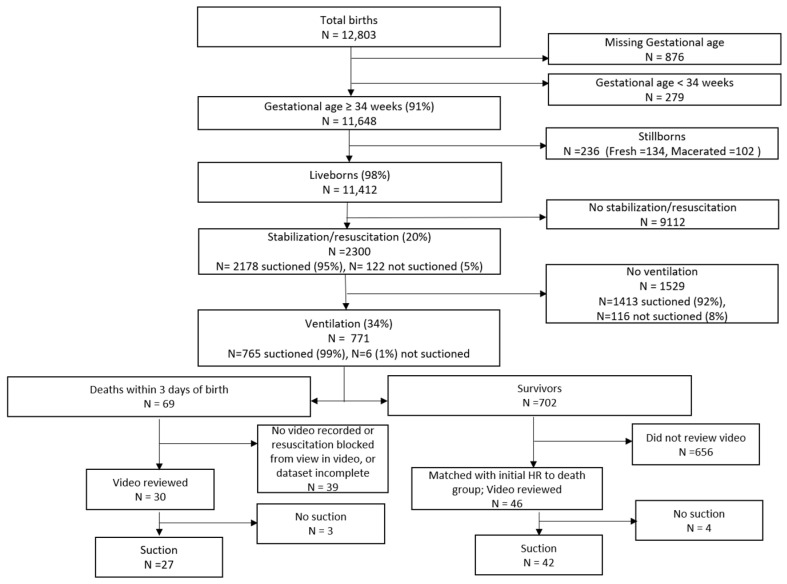
Cohort diagram.

Of the 12,803 newborns born in the study period, 11,648 had a recorded gestational age ≥34 weeks and 236 were stillborn (Figure 1). There were 11,412 liveborn newborns with a gestational age ≥34 weeks. Among these, a total of 2300 (20.2%) received stabilization interventions, 2178 (19.1%) were suctioned, and 771 (6.8%) were ventilated. Of the 76 included newborns, 69 were suctioned.

Table 1 compares the newborn characteristics and labor information of the 76 included newborns who died versus survived. Newborns who died had a lower birthweight and abnormal fetal HR both during labor and just prior to delivery compared to those who survived. Apgar scores assigned at 1 min and 5 min were lower for those who died compared to survivors. Table 2 compares resuscitation interventions, timelines, and newborn responses. The duration of resuscitation was longer, expired volume during BMV was higher, and duration from first to final ventilation was longer for newborns who died versus those who survived. There were no differences in suctioning characteristics between the two groups.

### 3.1. Suction Events and Heart Rate Changes

Sixty-nine resuscitations included at least one SE. A similar proportion of newborns who died (27/30, 90%) and survived (42/46, 91%) were suctioned. There were no significant differences in suctioning characteristics between those who survived compared to those who died, and bulb suction was mostly used in both groups (Table 2).

**Figure 2:**
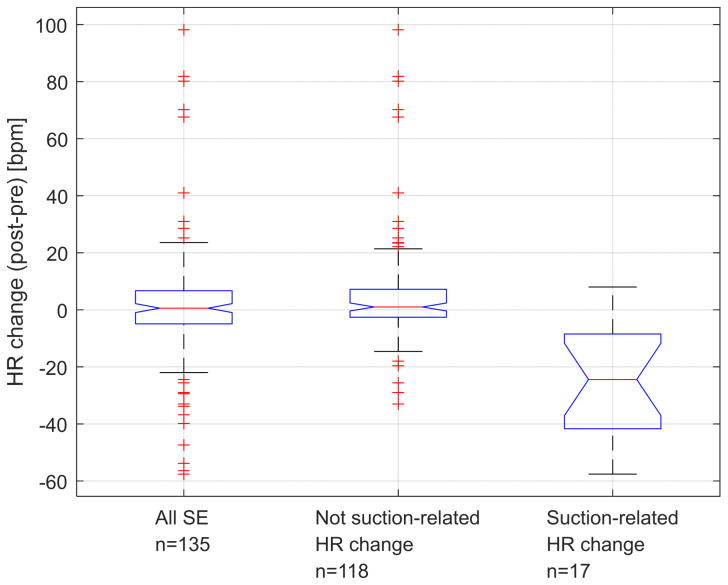
Box plots of all SEs, not suction-related HR changes, and suction-related HR changes in the 135 suction events. Outliers are marked in red.

**Figure 3:**
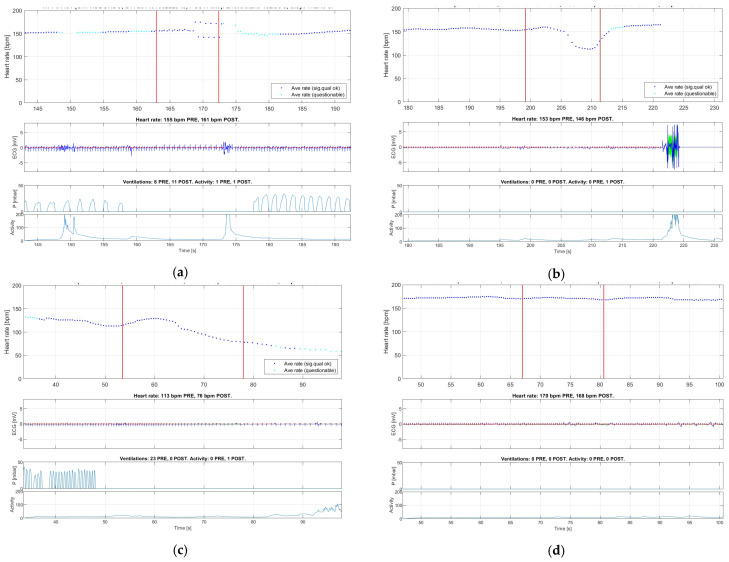
Illustrations of different types of changes in heart rate related to a suction event (annotated between the red horizontal lines in the upper panel): (**a**) arrhythmia (bigeminy); (**b**,**c**) brief or sustained fall in HR > 15%; and (**d**) no change in HR during suctioning. Red vertical lines indicate the start and stop of the suction event.

There were 135 SEs performed during 54 newborn resuscitations which had complete HR and ventilation data and were included in HR change analysis (Figure 2). Overall, there were no significant changes in HR during suctioning, with median “all” delta HR = 0.6 (quartiles −5.0, 6.8; *p* = 0.34). However, 17 SEs (12.6%) in 14 newborns were manually identified to have caused a suction-related HR change. This was defined as a fall in HR > 15% (*n* = 16) or an arrhythmia (*n* = 1). In this group, the median decrease in HR was 24 BPM with a delta HR = −24.4 (quartiles −43.6, −8.1; *p* < 0.001). Among the fourteen individual babies with at least one instance of suction-related HR change, nine died (9/14 = 64%). Among the 40 babies with no instances of suction-related HR changes, 11 died (11/40 = 28%; *p* = 0.014). Figure 3 shows an example of an arrhythmia (Figure 3a), two examples of fall in HR (Figure 3b,c), and an example of no suction-related HR change (Figure 3d).

Additionally, there were 11 SEs (8.1%) in seven newborns classified as a “HR change unrelated to suctioning”. In these cases, the cessation of PPV immediately prior to the initiation of suctioning led to a significant fall in HR (>30 BPM). Of the seven individual babies with at least one instance of “HR change unrelated to suctioning”, five died (5/7; 71%). Among the 47 babies with no instances of “HR change unrelated to suctioning”, 15 died (15/47, 32%, *p* = 0.043).

The upper panel illustrates heart rate in beats per minute (bpm). The middle panel illustrates ECG QRS complexes. The lower panel illustrates ventilation pressure.

The video recordings of the 17 SEs with suction-related HR changes were reviewed in more detail. Of the 17 SEs, 14 involved bulb suction and three involved catheter suction. Among the sixteen cases with a fall in HR, one occurred during a suction period of more than two minutes, and the entire head of the suction bulb was placed in the baby’s mouth. In another case, the baby was stimulated by tapping the feet or rubbing the back during suctioning. In two more cases, the baby received back stimulation and bulb suctioning concurrently. In three cases, a catheter was used and appeared to be inserted deep into the mouth. In the one case of arrhythmia, bulb suction was used.

### 3.2. Probability of Suction-Related HR Changes in Relation to Newborn Characteristics and Other Factors

**Figure 4:**
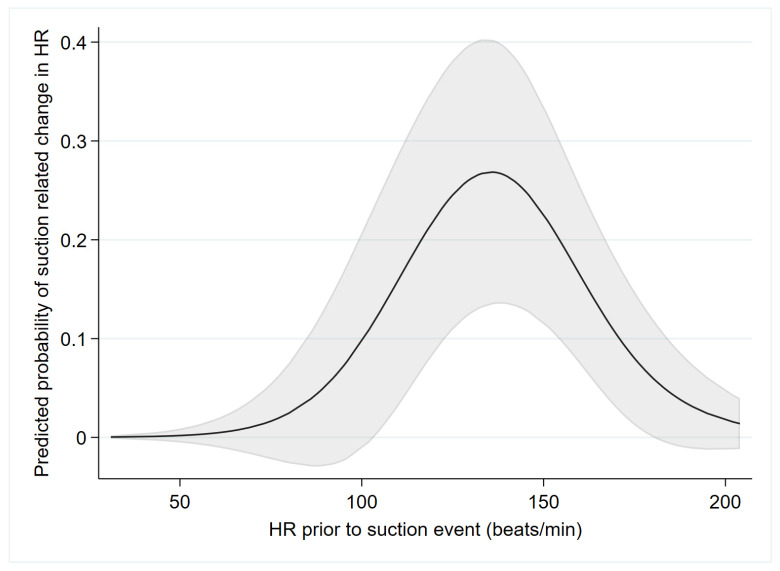
Predicted probabilities (with a 95% confidence band indicated by grey shaded area) of suction-related HR changes as a function of HR prior to starting suction.

Table 3 presents factors analyzed for their potential association with suction-related HR changes. The HR prior to SE was the only variable found to be statistically significantly associated with a suction-related change in HR. The predicted probability of suction-related HR change as a function of HR prior to starting the SE is presented in Figure 4. A suction-related change in HR was more likely with prior HR around 100–170 BPM. A suction-related change in HR was less likely for both prior HR lower than 100 BPM and higher than 170 BPM. Additional predicted probabilities of suction-related changes in HR variables, which were not found to be statistically significantly associated with HR changes, are presented as Supplementary Materials (Figure S1).

Results were obtained by multilevel logistic regression with restricted cubic splines (3 knots), including 135 observations of 54 infants. Heart rate prior to suction event was found to be statistically significantly associated with the outcome (see Table 3).

## 4. Discussion

In this study, 95% of newborns receiving stabilization/resuscitation were suctioned. These are higher rates than reported in previous studies [2,3]. Significant suction-related HR responses (brief/sustained fall in HR or arrhythmia) were observed in 13% of suction events, with the majority of these cases suctioned with bulb suction. Interestingly, there were also 11 SEs (8.1%) with no suction-related HR change, where the cessation of PPV and/or stimulation immediately prior to the initiation of suctioning led to a significant fall in HR.

Current guidelines recommend suctioning only if there is a suspected obstructed airway [13,17,18,21]. The 69 newborns who were suctioned in this study had a median of two SEs per resuscitation, with a median total suctioning duration of 32 s. Additionally, for each SE, there was a median of 3.6 suction insertion attempts. This suggests that suctioning was not performed just to clear an initial airway obstruction. In this study, health care providers had undergone HBB training, yet suctioning was performed multiple times in a resuscitation, possibly delaying the initiation or continuation of ventilation or other interventions [19].

Various risks associated with suctioning have been reported. In a randomized trial including 140 normal-term newborns, those in the suction group took longer to reach higher SpO2 levels [6]. In a smaller controlled study of 30 normal term newborns, the suction group took longer to reach higher SpO2 levels and had significantly lower SpO2 through the first 6 min of life [7]. In a study of 35 premature newborns, those newborns that were suctioned had increased intracranial pressure noted during the procedure [22]. Newborns receiving more than two oral suctioning procedures in the neonatal intensive care unit have a higher risk of staphylococcal colonization and sepsis [21].

For the first time, we are able to describe potential suction-related HR changes during ongoing resuscitation of near-term and term asphyxiated newborns in the delivery room. We document that almost 13% of SEs induced a substantial fall in HR. In another 8.1% of SEs, a significant decrease in HR was observed due to the interruption of ventilation. No differences were found in suctioning characteristics (i.e., number, length, or duration of suctioning events, see “suctioning during resuscitation” in Table 2) between newborns who died versus those who survived. However, significantly more babies (64%) with at least one instance of suction-related HR change died compared to babies (28%) with no instances of suction-related HR changes. This is an interesting observation, given the fact that most cases of a suction-related decrease/arrythmia in HR occurred in babies with a prior suction HR within the “normal range”. While we cannot conclude on the impact of this on mortality based on our data, these observations are consistent with Linde et al. [10] who found that a sudden and significant decrease in HR during ongoing resuscitation was associated with an increased risk of death.

Other studies did not find differences in outcomes on suctioned newborns. The 2018 Cochrane review on routine oro/nasopharyngeal suctioning versus no suctioning at birth reported no differences in newborn mortality, need for resuscitation, admission to neonatal intensive care, and five-minute Apgar score [23]. In a randomized equivalency trial in a United States urban hospital, which included apneic newborns ≥35 weeks of gestation, no differences in clinical outcomes were seen between those suctioned or wiped at the mouth [24]. In a prospective randomized trial of 84 healthy term newborns delivered by cesarean section, suctioning (oro/nasopharyngeal) did not affect oxygen saturation (SpO2) and HR in the first hour after birth [25].

Suction-related HR changes were observed during 17 SEs, involving 14 individual newborns. Overall, in this group, there was a median decrease in HR of 24 BPM. The majority of suction-related HR changes (14/17) were observed when using a bulb suction device. In contrast, in a study comparing newborns suctioned with a bulb or catheter suction, those suctioned with a bulb showed no decreases in HR, while some of those suctioned with a catheter developed arrhythmias or became apneic [26]. The limited use of catheter suction in our study explains the low number of catheter suction-related HR changes.

In our study, newborns with HR prior to suctioning within the “normal range” had a greater risk of suction-related HR changes. While the mechanism of the HR changes remains unclear, we speculate that these newborns could have been particularly prone to vagal stimulation, due to the stimulation of the posterior pharynx. Non-breathing hypoxic newborns with a HR above 170 bpm were probably catecholamine-stressed and less likely to be influenced by vagal stimulation. Asphyxiated newborns with a HR below 100 bpm may have had impaired reflexes. Nevertheless, our findings should serve as an important reminder to avoid unnecessary airway suctioning in all babies.

There are limitations to this study. First, the data set is small, with little power to compare deaths versus survivors. The videos only captured the resuscitation table and additional suctioning may have occurred off the resuscitation table without our knowledge. The criteria used for selecting the study population may limit the generalizability of the findings. In addition, there were only a few cases of suction catheter use during resuscitations, and thus there is little statistical power to detect differences in risk of suction catheter-related changes in HR as compared to the use of bulb suction. Suction depth, which may have been a possible predictor for suctioning HR-related changes, was not assessed. This study was performed in a low-resource setting and its findings may not be applicable to higher-resource settings.

The greatest strength of our study is the unique comprehensive research infrastructure capturing resuscitation activities including suctioning in the delivery room, with observational data collected by trained research assistants and objective data collected by video recordings and biomedical ventilation and HR data. Each study video was reviewed by two independent annotators, a clinician and engineer, following an annotation protocol.

## 5. Conclusions

Suctioning was performed more frequently than recommended by the current guidelines or by guidelines at the time of the study. There were no differences in the suction characteristics of newborns who died versus those who survived. However, in 13% of SEs, a significant HR change (decrease in HR or arrhythmia) was observed in relation to the suctioning. This represents a potential additional harm to already depressed newborns undergoing resuscitation, and more babies with a suction-related HR change died compared to those with no HR changes. Continuous training of staff emphasizing the importance of limiting suctioning is necessary.

## Figures and Tables

**Table 1:** Comparison of newborn and labor characteristics among resuscitated newborns who died versus survived.

Characteristics	Total *n* = 76	Deaths *n* = 30	Survivors *n* = 46	p ^1^	p ^2^
Birth weight (grams)	3060 ± 560	2841 ± 491	3203 ± 561	**0.004**	**0.009**
Gestational age (weeks)	38.1 ± 1.6 *^n^* ^= 72^	37.9 ± 1.4 *^n^* ^= 27^	38.2 ± 1.6 *^n^* ^= 45^	0.35	0.47
Female	26 (34%)	13 (43%)	13 (28%)	0.18	0.26
Abnormal FHR on admission	2 (3%) *^n^* ^= 66^	1 (4%) *^n^* ^= 24^	1 (2%) *^n^* ^= 42^	0.69	0.56
Cervical dilatation on admission (cm)	6.3 ± 2.4 *^n^* ^= 64^	6.2 ± 2.6 *^n^* ^= 22^	6.4 ± 2.3 *^n^* ^= 42^	0.84	0.96
Fetal presentation				0.60	0.49
Cephalic	67 (88%)	26 (87%)	41 (89%)		
Breech	6 (8%)	2 (7%)	4 (9%)		
Others (transverse, prolapse)	3 (4%)	2 (7%)	1 (2%)		
Abnormal FHR during labor	9 (15%) *^n^* ^= 62^	7 (30%) *^n^* ^= 23^	2 (5%) *^n^* ^= 39^	**0.007**	**0.010**
Amniotic fluid ^5^	(*n* = 75)		(*n* = 45)	0.22	0.37
Clear	39 (52%)	12 (40%)	27 (60%)		
Slight meconium	11 (15%)	6 (20%)	5 (11%)		
Thick meconium	24 (32%)	12 (40%)	12 (27%)		
Blood stained	1 (1%)	0 (0%)	1 (2%)		
Abnormal final FHR before delivery	9 (14%) *^n^* ^= 64^	7 (29%) *^n^* ^= 24^	2 (5%) *^n^* ^= 40^	**0.008**	**0.010**
Multiples (twins)	3 (4%)	1 (3%)	2 (4%)	0.82	0.60
Labor complications ^6^	6 (8%)	2 (7%)	4 (9%)	0.75	0.93
Mode of delivery				0.53	0.85
Spontaneous vaginal delivery	41 (54%)	14 (47%)	27 (59%)		
Cesarean section	32 (42%)	15 (50%)	17 (37%)		
Assisted breech delivery	3 (4%)	1 (3%)	2 (4%)		
Apgar score assigned by attending midwife					
1 min	7 (4, 7)	4 (3, 5)	7 (7, 8)	**<0.001 ^3^**	**<0.001 ^3^**
5 min	10 (8, 10)	9 (6, 10)	10 (10, 10)	**<0.001 ^3^**	**<0.001 ^3^**
First newborn HR (bpm)	77 (55, 139)	66 (52, 94)	94 (59, 154)	0.098 ^4^	

Data presented as mean ± SD, count (%), or median (quartiles). Number of observations is indicated if missing data. Likelihood ratio test *p*-values from regression analyses without (p ^1^) and with (p ^2^) adjustment for first HR, using linear regression for symmetric continuous variables, binary or multinomial logistic regression for categorical variables, except ^3^ log-linear regression with robust standard errors for reversed left-skewed variables (Wald test *p*-values), and ^4^ quantile regression for right-skewed variable (Wald test *p*-values). Bolded *p*-values indicate *p* < 0.05. ^5^ Thick, combined with blood, stained for regression analysis. ^6^ Labor complications = bleeding, eclampsia, obstructed labor, shoulder dystocia. FHR = fetal heart rate, HR = heart rate, bpm = beats per minute, cm = centimeter.

**Table 2:** Comparison of resuscitation interventions, timelines, and newborn responses among resuscitated newborns who died versus survived.

Characteristics	Total *n* = 76	Deaths *n* = 30	Survivors *n* = 46	p ^1^	p ^2^
*General*					
Duration of resuscitation	388 (223, 602)	609 (336, 1218)	288 (182, 438)	**0.003 ^3^**	**0.013 ^3^**
Time from birth to cord clamping (sec)	33 (15, 57) *^n^* ^= 74^	19 (14, 50) *^n^* ^= 29^	35 (12, 58) *^n^* ^= 45^	0.10 ^3^	0.69 ^3^
Time from birth to placement of ECG sensor (sec)	107 (71, 142) *^n^* ^= 72^	115 (71, 131) *^n^* ^= 28^	103 (71, 166) *^n^* ^= 44^	0.50 ^3^	0.42 ^3^
Time from birth to start ventilation (sec)	108 (76, 158) *^n^* ^= 74^	125 (81, 146) *^n^* ^= 29^	104 (73, 176) *^n^* ^= 45^	0.32 ^3^	0.63 ^3^
Duration from first to final ventilation (sec)	174 (104, 344) *^n^* ^= 72^	557 (208, 700) *^n^* ^= 28^	122 (77, 192) *^n^* ^= 44^	**<0.001 ^3^**	**<0.001 ^3^**
HR at start of first ventilation (bpm)	82 (61, 133) *^n^* ^= 58^	61 (54, 79) *^n^* ^= 22^	121 (69, 160) *^n^* ^= 36^	**0.002 ^3^**	0.81 ^3^
HR at cessation of ventilation (bpm)	150 (129, 167) *^n^* ^= 70^	142 (123, 155) *^n^* ^= 25^	156 (140, 171) *^n^* ^= 45^	0.096 ^3^	0.31 ^3^
HR increase from first to final ventilation (bpm)	27 (2, 82) *^n^* ^= 52^	63 (9, 92) *^n^* ^= 17^	25 (1, 74) *^n^* ^= 35^	0.085 ^3^	0.40 ^3^
*Ventilation Parameters*					
Inflated volume (ml) ^4^	39 (25, 49)	36 (25, 46)	41 (25, 51)	0.48 ^3^	0.47 ^3^
Mask leakage (%) ^4^	50 ± 21	47 ± 22	52 ± 20	0.39	0.50
Expired volume (ml) ^4^	14 (8, 25)	16 (7, 25)	13 (8, 26)	0.35 ^3^	0.37 ^3^
Expired volume (ml/kg) ^4^	6.2 (3.7, 10.1)	9.7 (3.8, 11.9)	4.8 (3.3, 8.2)	**0.001 ^3^**	**0.001 ^3^**
Ventilation frequency (per minute) ^4^	52 (41, 68)	51 (41, 67)	54 (40, 68)	0.64 ^3^	0.84 ^3^
Peak inflation pressure (mBar) ^4^	34 ± 8	33 ± 7	34 ± 8	0.43	0.56
Expired CO_2_ (%) ^4^	3.2 (1.8, 4.2)	3.1 (1.3, 3.8)	3.5 (2.1, 4.4)	0.57 ^3^	0.57 ^3^
*Suctioning during Resuscitation*					
Resuscitation period analyzed for suctioning (sec)	391 (245, 420)	420 (360, 471)	312 (213, 416)		
Babies with at least one SE	69 (91%)	27 (90%)	42 (91%)	0.85	0.86
Number of SEs per baby	2 (1, 4)	2 (1, 4)	2 (1, 4)		
Total duration of suctioning (sec)	32 (15, 64)	32 (18, 67)	32 (13, 61)		
Proportion of time spent suctioning (%)	10 (5, 19)	10 (4, 17)	11 (6, 20)		
Infants receiving at least one catheter suction (vs. only bulb suction or no suction)	17 (22%)	8 (27%)	9 (20%)	0.47	0.67
Number of suction insertions per baby	9 (5, 16)	9 (6, 15)	11 (5, 18)		
Number of mouth suction insertions	6 (3, 9)	5 (3, 8)	6 (3, 11)		
Number of nose suction insertions	4 (2, 7)	4 (2, 7)	4 (2, 7)		
Number of suction insertions/SE ^5^	3.6 (3.0, 5.4) *^n^* ^= 68^	3.4 (2.4, 6.0) *^n^* ^= 26^	3.9 (3.0, 5.1) *^n^* ^= 42^		
Number of mouth suction/SE	2.0 (1.5, 3.0)	1.8 (1.3, 3.1)	1.3 (1.0, 2.0)		
Number of nose suctions/SE	1.5 (0.8, 2.1)	1.6 (0.6, 2.6)	1.3 (1.0, 2.0)		

Data presented as mean ± SD, count (%), or median (quartiles). Number of observations is indicated if missing data. Likelihood ratio test *p*-values from regression analyses without (p ^1^) and with (p ^2^) adjustment for first HR, using linear regression for symmetric continuous variables and binary or multinomial logistic regression for categorical variables, except ^3^ quantile regression for right-skewed variables (Wald test *p*-values). Bolded *p*-values indicate *p* < 0.05. Sec = seconds, HR = heart rate, bpm = beats per minute, SE = suction event. ^4^ Reported as mean value for each newborn resuscitation. ^5^ Including only babies with at least one SE.

**Table 3:** Assessment of potential predictors of suction-related changes in heart rate.

Predictors	Suction-Related Change in HR (*n* = 17)	No Suction-Related Change in HR (*n* = 118)	OR ^1^ (95% CI)	p ^2^	p_NL_ ^4^
*Categorical*					
Female	6 (35%)	26 (22%)	1.93 (0.51–7.33)	0.33	
Amniotic fluid		(*n* = 115)		0.10	
Clear	6 (35%)	61 (53%)	1 (ref)		
Slight	2 (12%)	14 (12%)	1.45 (0.26–8.15)	0.67	
Thick/Blood	9 (53%)	40 (35%)	2.29 (0.69–7.53)	0.17	
Catheter (vs. bulb)	3 (18%)	32 (27%)	0.58 (0.16–2.02)	0.39	
Ventilation prior to SE	8 (47%)	60 (51%)	0.86 (0.28–2.66)	0.79	
Stimulation prior to SE	13 (76%)	75 (64%)	1.86 (0.56–6.16)	0.31	
*Continuous*					
Gestational Age (weeks)	39 (38, 39) *^n^* ^= 15^	38 (36, 39) *^n^* ^= 114^	1.37 (0.98–1.93)	0.066	0.095
Birthweight (g)	2780 (2530, 3320)	3100 (2730, 3350)		0.080 ^3^	**0.043**
First observed HR (bpm)	74 (58, 123)	72 (52, 131)	0.96 (0.85–1.07)	0.45	0.089
Time post delivery to initiation of SE (sec)	113 (64, 160)	103 (54, 183)		0.12 ^3^	**0.047**
HR prior to SE (bpm)	144 (129, 155)	137 (89, 170)		**0.003 ^3^**	**0.001**
Duration of SE (sec)	18 (10, 23)	14 (7, 23)	1.02 (0.99–1.04)	0.14	0.98

There were 54 infants in the sample (20 deaths). Some infants contributed more than one observation. ^1^ Odds ratios (OR) with 95% confidence interval (CI) for suction-related changes in HR from logistic regression allowing for clustering of suctioning events within infants, given per 100 g for BW, per 10 bpm for first-observed HR, and per 10 sec for time post delivery. ^2^ *p*-value from Wald test of overall effect of variable (^3^ while allowing for non-linear effect), ^4^ *p*-value from Wald test of non-linear effect of continuous variable. The estimated effect of HR prior to SE is illustrated in Figure 4. Sec = seconds, HR = heart rate, bpm = beats per minute, SE = suction event.

## Data Availability

The data presented in this study are available on reasonable request to the corresponding author. However, we are not allowed to make these openly available due to regulations from the National Institute of Medical Research in Tanzania.

## Supplementary Materials

The following supporting information can be downloaded at: https://www.mdpi.com/article/10.3390/children10091540/s1, Figure S1: Predicted probabilities of suction-related changes in infants’ heart rate.
