# Supplementary material for: Use of Suctioning during Newborn Resuscitation and Its Effects on Heart Rate in a Low-Resource Setting, Tanzania
Source: Children (Basel). 2023 Sep 12;10(9):1540. doi: 10.3390/children10091540 (PMC10529279; doi:10.3390/children10091540)
Supplement: Supplementary file 1 [file children-10-01540-s001.zip › children-2596666-supplementary.pdf]

Supplementary Materials

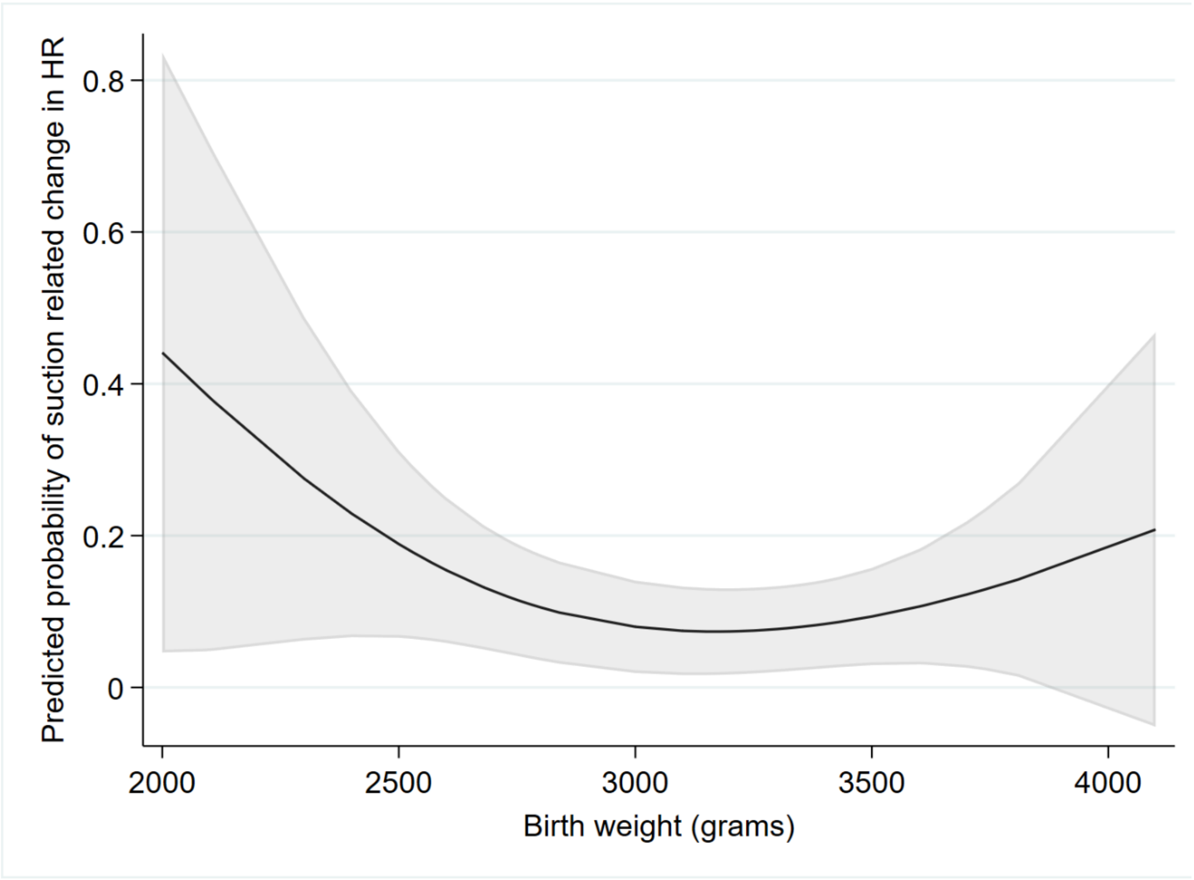

(a)

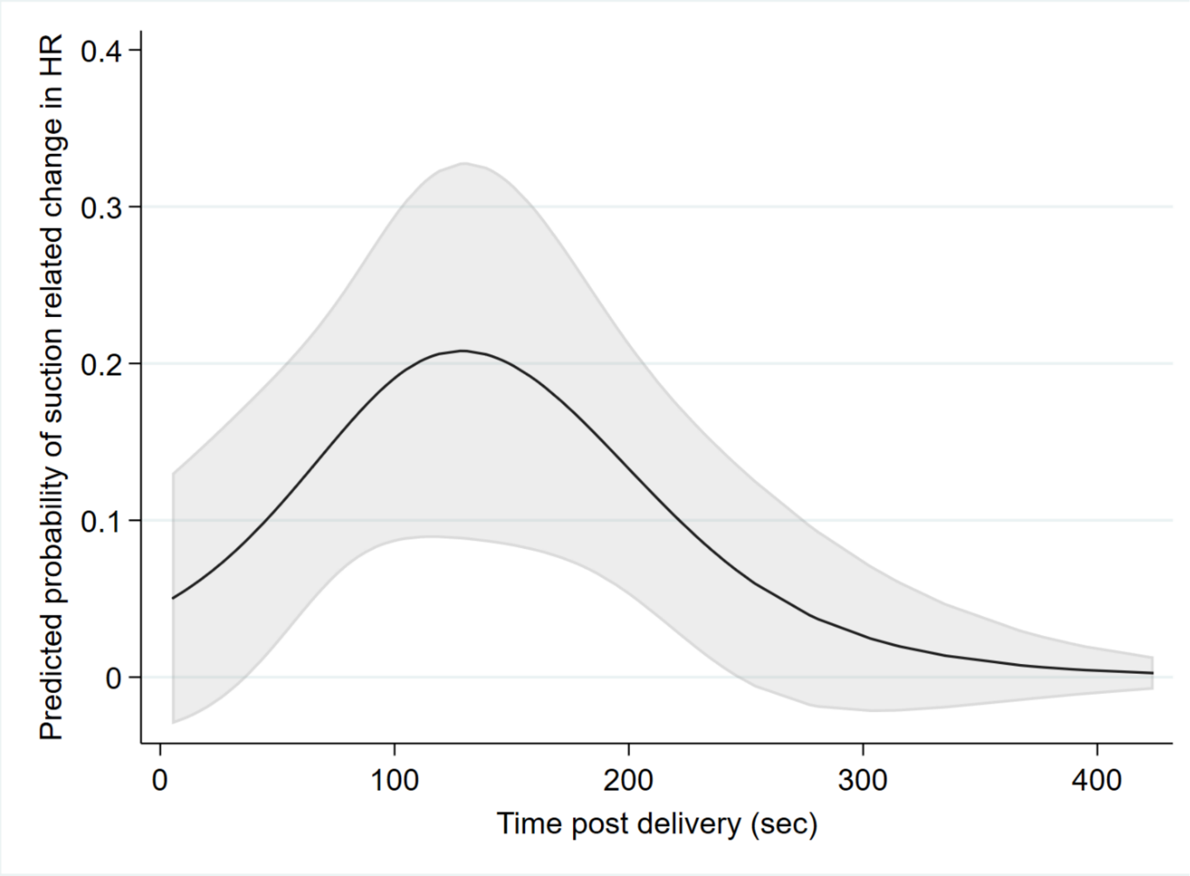

(b)

Figure S1: Predicted probabilities of suction related changes in infants' heart rate.

Predicted probabilities of suction related change in heart rate (HR) (with 95% confidence bands indicated by grey shaded areas) as a function of (a) birth weight; (b) time post delivery to initiation of SE (sec). Results obtained by multilevel logistic regression with restricted cubic splines (3 knots) including 135 observations of 54 infants. These variables were not found to be statistically significantly associated with the outcome (see Table 3).
